# Preferences, educational messaging, and demand creation channels for multipurpose-prevention technologies (MPTs) among women in South Africa

**DOI:** 10.1186/s12889-023-16904-0

**Published:** 2023-10-25

**Authors:** Paballo Mataboge, Nqaba Mthimkhulu, Alison Kutywayo, Catherine E. Martin, Mbali Mazibuko, Khanyiswa Kwatsha, Nthabiseng Makalela, Elmari Briedenhann, Vusile Butler, Rutendo Bothma, Saiqa Mullick

**Affiliations:** Wits RHI, Faculty of Health Sciences, University of the Witwatersrand, Johannesburg, South Africa

**Keywords:** Multipurpose-prevention technology, HIV prevention, Contraception, Demand creation, South Africa, Women

## Abstract

**Background:**

South African women, including female sex workers (FSWs), are disproportionately affected by HIV, experience a high unmet need for contraception and high rates of sexually transmitted infections (STIs). Products that address the interlinked risk between HIV, unplanned pregnancy, and other STIs are needed. There are several multipurpose-prevention technologies (MPTs) in development, aimed at preventing both HIV and pregnancy. This study aimed to explore educational messaging and demand creation channels to improve the potential uptake of a hypothetical MPT implant, using participatory action research. It also aimed to look at product and service provision attributes preferred by potential end users.

**Methods:**

Between July and August 2022, 303 PrEP-eligible adolescent girls and young women (AGYW) (18–24 years), women > 24 years, and FSW’s (≥ 18 years) participated in 4-hour workshops, where they were asked about their ideal messaging and demand creation channels and their preferences for different attributes of an MPT implant. Quantitative descriptive analysis was conducted to determine the frequency and ranking for each demand creation message, channel, and each product and service provision attribute, by population group. A chi-square test was used to assess MPT implant characteristics associated with age. Qualitative data were analysed using deductive and thematic analysis.

**Results:**

A total of 104 AGYW, 157 women > 24 years, and 42 FSWs participated in the PAR workshops. Participants preferred demand creation messages that were empowering, motivational and encouraged body autonomy. The use of slang was popular. Community radio stations and newspapers, social media, and information at hospitals and clinics were participants’ preferred MPT demand creation channels because they were easily accessible. Providing long-term and dual HIV and pregnancy protection, receiving implant services at the local clinic, manageable side effects, discreet and private implant, and numbing the area before insertion and removal, were the most preferred product and service provision attributes.

**Conclusion:**

Early considerations for women’s product preferences are key to product development. Educational messaging around the MPT implant should be empowering and in local languages, this may motivate women to learn more about it and use it. Multiple demand creation channels should be used to engage both young and older populations, which may ensure better reach.

**Supplementary Information:**

The online version contains supplementary material available at 10.1186/s12889-023-16904-0.

## Introduction

Women are disproportionately affected by HIV in South Africa, particularly adolescent girls and young women (AGYW) and female sex workers (FSW) [1]. The prevalence of HIV is estimated to be at 10.9% among AGYW, 27.5% for women aged 25–29 years [1], and 61.9% among FSWs [2]. Early sexual debut, low condom use, having multiple sexual partners, transactional sex and substance use increase HIV risk among women, including FSWs [1, 3–7]. Harmful cultural and gender norms, gender-based violence, economic gender inequality, and age-disparate relationships limit women’s ability to practice safe sex and therefore increase their HIV risk [7–13]. There is also a high unmet need for contraception among women in South Africa [14] and high rates of sexually transmitted infections (STIs) [15]. In the 2016 South Africa Demographic Health Survey, the unmet need for contraception among adolescent girls (15–19 years) was 8.0%, and young women (20–24 years) (16.0%) [14]. A Unitaid-funded PrEP Project working across 12 South African sites, found that almost a third (27.7%) of AGYW in their study were not using contraception at their first visit when they initiated on oral pre-exposure prophylaxis (PrEP) for the prevention of HIV [16]. In 2019, there were also high rates of unintended pregnancies nationally, estimated to be at 76.3% among AGYW (15–19 years), 56.5% among young women (20–24 years), 44.1% among women 25–34 years and 45.2% among older women (35–49 years) [17]. The estimated prevalence of STIs among clinic-going AGYW in South Africa ranged from 8.0-20.6% for chlamydia, and 1.4–8.9% for gonorrhoea [18].

Acknowledging the interlinked risks of HIV and STI acquisition and unplanned pregnancy, multipurpose-prevention technologies (MPT) are currently being developed [19]. Condoms are the only MPT currently available, preventing pregnancy, HIV, and STIs [19]. Despite being readily available, condoms are not feasible for many women due to the unequal power dynamics, challenges with negotiating condom use, and sexual preferences which may limit a female’s ability to consistently use a condom [19, 20]. However, in the last decade, there has been a growing array of new MPTs in development, with over two dozen in active development [19]. Most MPTs are still in the early pre-clinical to clinical phase, with each adopting a different delivery method (oral pills, vaginal rings and films, long acting injectables, transdermal compounds and implants) [19]. MPTs have the potential to improve the effective use of PrEP and contraception, eliminating the need for multiple, separate clinic visits for family planning and other sexual and reproductive health (SRH) services [21]. They also have the potential to provide discretion, reduce stigma, reduce user and health system burden, and offer cost effective prevention options [22–24]. The hypothetical MPTs of interest in this study were the one-year or two-year non-biodegradable, biodegradable, and refillable subcutaneous implants, providing simultaneous prevention against HIV and pregnancy. These MPTs are not currently available.

When introducing new prevention products, it is vital to involve potential end-users in their design to ensure they meet their needs, align with their preferences, and are implemented in ways that will allow maximum effect and public health benefit [22]. Strategic communication approaches that appeal to end-user needs, hopes, and preferences are key to creating demand [25]. One way to achieve this engagement is through participatory action research (PAR), a qualitative research methodology involving researcher/participant collaboration to understand social issues from the participant’s perspective, seeking to impart social change [26]. Through PAR, the participants’ feelings and views are revealed without researcher manipulation, as the participant actively makes informed decisions throughout the research process [26].

We aimed to explore educational messaging and content, and demand creation channels that could improve the uptake of hypothetical MPT implants among AGYW, women > 24 years, and FSWs, using a PAR approach. We also aimed to explore the preferences for prevention product attributes of the following hypothetical MPTs; the one-year or two-year non-biodegradable, biodegradable, and refillable subcutaneous implants, providing simultaneous prevention against HIV and pregnancy.

## Methods

This study aimed to investigate the potential uptake of MPT implants among PrEP eligible clients in South Africa between July and November 2022, as a sub-component of a study funded by the Bill and Melinda Gates Foundation. The main study comprised of an information session, workshops with participatory action activities and a self-administered survey among AGYW, women > 24 years, FSW ≥ 18years, and men 18–40 years. The study also comprised healthcare provider workshops with PAR activities and in-depth interviews with key informants. This paper only reports findings from the workshops conducted between July and August 2022 with AGYW, women > 24 years, and FSWs.

### Study design and setting

We conducted a cross-sectional mixed methods research study, consisting of PAR workshops and a survey, in three districts in three South African provinces (Tshwane District, Gauteng Province; OR Tambo District, Eastern Cape Province, and King Cetshwayo District (KCD), KwaZulu-Natal Province). The three sites represent different geographies (urban, peri-urban, and rural), providing diverse social contexts. In Tshwane District, recruitment took place in and around two rural residential townships namely, Soshanguve and Ga-Rankuwa, specifically within two fixed clinics and a linked mobile clinic. Tshwane had an antenatal HIV prevalence of 23.1% in 2019 [27]. Recruitment of self-identifying FSWs was undertaken at a Wits RHI-supported Key Populations clinic and linked mobile clinic in Tshwane. In the OR Tambo district, participants were recruited from two clinics in Mthatha, with an antenatal HIV prevalence rate of 35.2% in 2019 [27]. In KCD, recruitment took place in four clinics across two sub-district: (Umfolozi:2) and (uMlalazi:2), with an antenatal HIV prevalence of 35% in 2019 [27]. All sites have been supported by Wits RHI to introduce oral PrEP through implementation science projects.

### Study population

The population groups of interest were AGYW (18–24 years), women > 24 years, and FSW (≥ 18 years). Women accessing health services at recruiting facilities or community outreach sites were eligible for participation if they were female, ≥ 18 years old, eligible for PrEP (self-reported HIV-negative), and willing and able to consent to participation in a 4-hour workshop which included an information session, PAR activities and a self-administered survey.

### Sampling and recruitment

The participants were recruited by trained fieldworkers and peer educators. Two approaches to recruitment were used: consecutive sampling of clients accessing SRH services at study sites, and snowball sampling through already recruited participants. The study employed consecutive sampling to recruit clients at study sites, whereby fieldworkers approached clients when they accessed SRH services, assessed their eligibility, gave a brief overview of the study procedures, and invited them to be enrolled in the study. Recruitment flyers were given to participants willing to take them home to enable the recruitment of eligible family members or friends through snowballing. The flyer contained the study contact number allowing participants to call the study team for further information. In Tshwane, FSWs accessing services at the Key Populations mobile outreach community sites and the fixed Key Populations primary healthcare clinic were recruited by the Key Populations’ peer educators.

### Study procedures

Participants attended a scheduled workshop of approximately 4-hours which was held at a local hotel or community venue, convenient for participants. Four workshops were conducted in each study district, two with AGYW participants and two with women > 24 years. In Tshwane, two additional workshops were held with FSWs. In total, 14 workshops were conducted. Each workshop was led by study staff, trained in study procedures and PAR activities and observed by at least two of the trained study team, using a workshop guide. For the FSW workshops, two FSW peer educators were also present to welcome participants and observe. All the PAR activities were designed and predetermined by the study team (KK, NM, EB) guided by the study objectives.

The workshop guide acted to guide the study team through the workshop proceedings, including questions to be posed to participants, and sought to capture the discussion points, questions, and reflections, as well as document the PAR activities. As partial participants [28], observers took part in the interactions of the workshop but not in the specific PAR activities. In these communities, there was a sufficient understanding of English therefore, the workshops were facilitated in English. Where necessary, the facilitator provided on the spot translations. Participants were also able to share their views using their local language, which the research team had a good understanding of. The study team welcomed the workshop participants with an icebreaker. They conducted an information session, educating participants on existing and new HIV prevention methods, the various MPTs in pre-clinical and clinical development, and a hypothetical MPT implant. The team explained the benefits, duration of use, administration method, side effects, and current or anticipated availability for each existing and future HIV prevention method. Where possible, a picture of the HIV prevention method was also included. Participants were then taken through PAR activities. Each of the activities is described in detail below:

### *Messaging and demand creation elections*

**Fig. 1:**
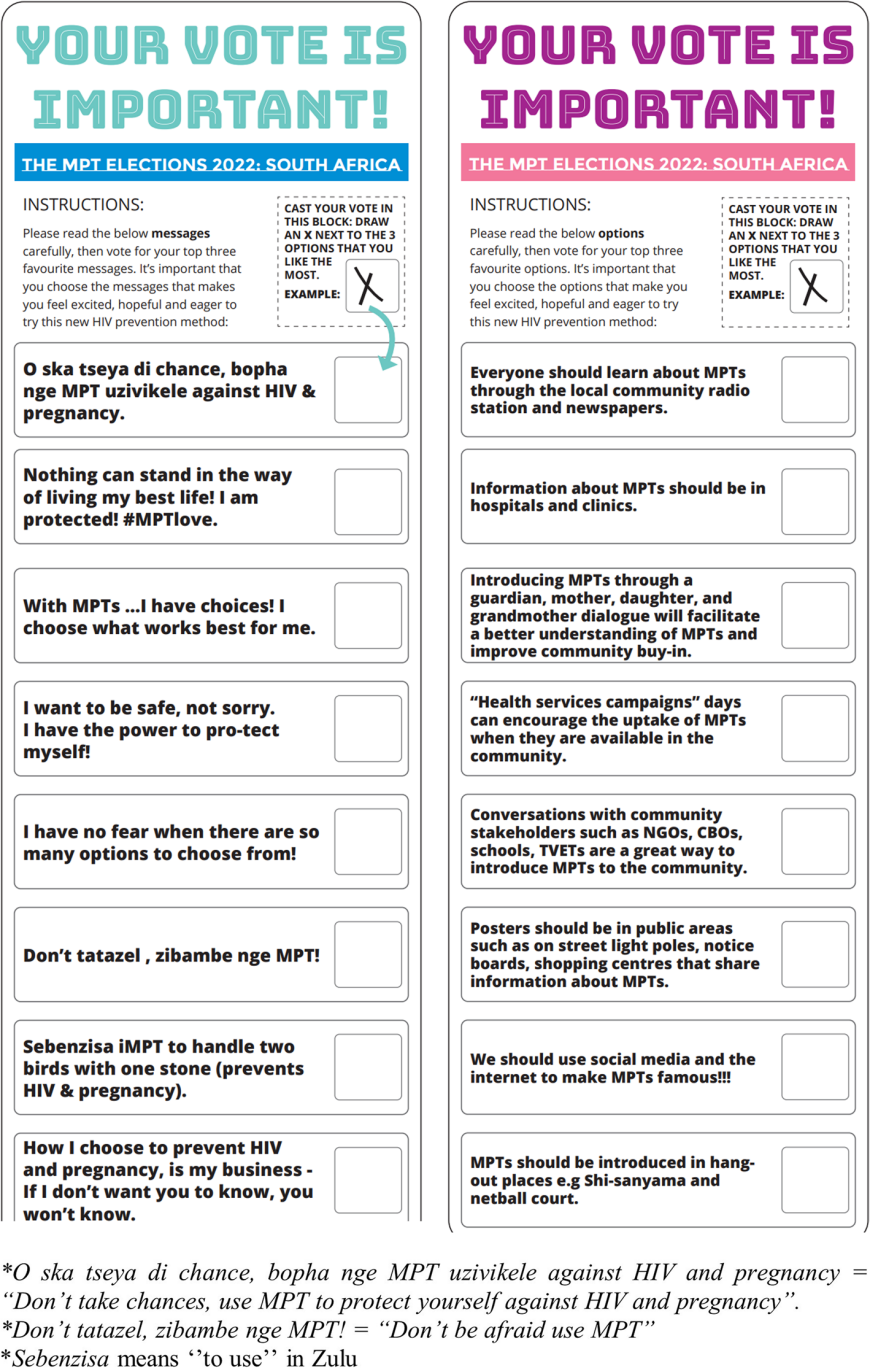
Ballot papers; Demand creation messages and channels

 The target audience for this activity was AGYW, women > 24 years, and FSWs, seeking to gain a deeper understanding of, and build evidence for developing demand creation and social mobilization approaches for future MPT implants. The room was set to simulate a real voting experience, with a voting booth, a voting box, and posters. The participants were each given two ballot papers – one to vote for their top three demand creation key messages and one to vote for their top three demand creation channels, and tactics (See Fig. 1). Once the participants had voted, the results for each ballot were captured and presented to the participants and a facilitated group discussion ensued. The discussion was guided by questions and discussion points on the workshop guide.

### ***Build your own MPT pizza***

**Fig. 2:**
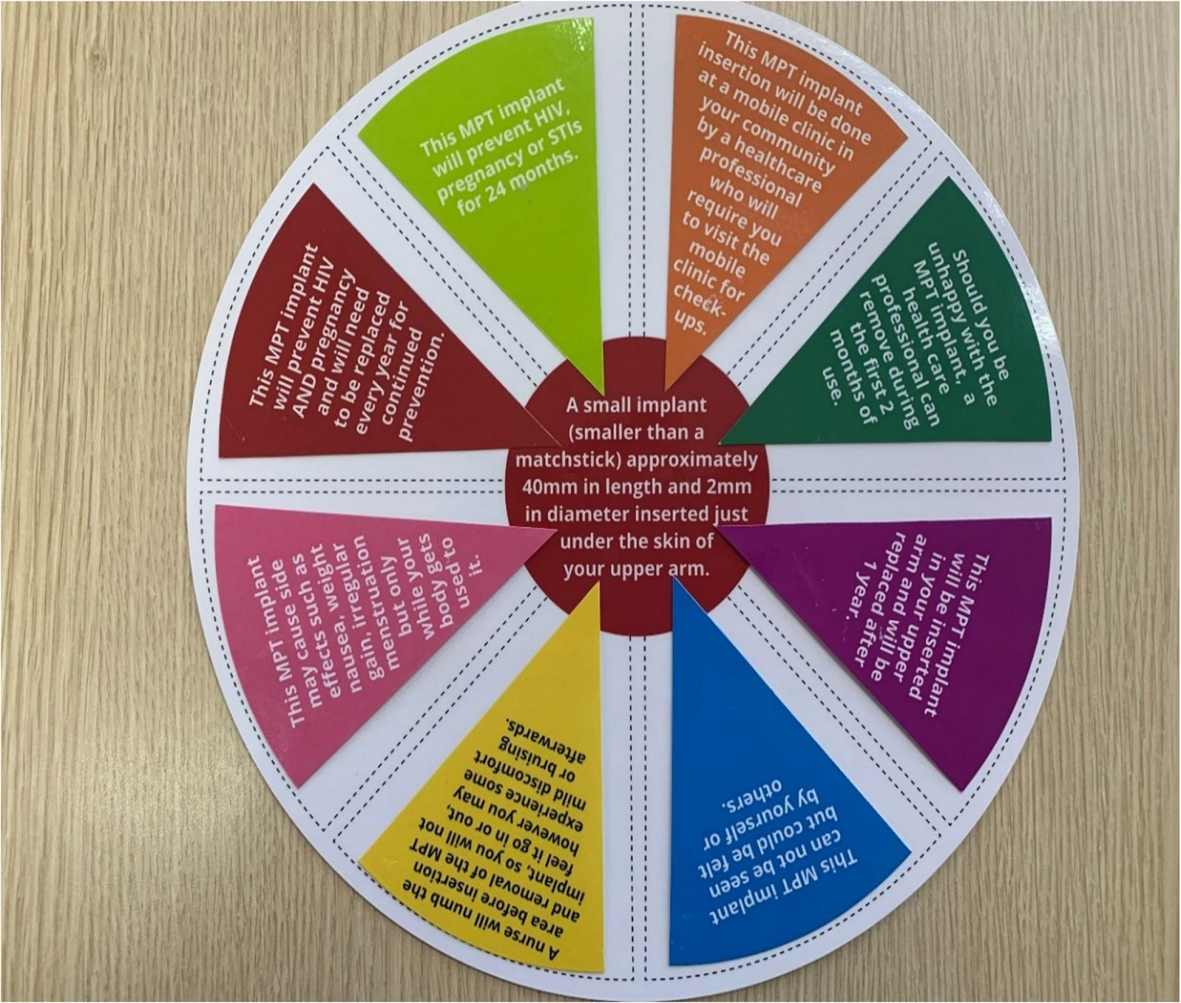
A picture of an ideal MPT product and service provision

 The target audience for this activity was AGYW and women > 24 years, seeking to understand preferred MPT implant characteristics. During this activity, each participant was given a laminated *‘pizza base’* and 24 *‘pizza pieces’.* Each pizza piece was colour coded according to a different MPT implant attribute; body placement, prevention characteristics (i.e., mono, or dual prevention), side effects, service delivery point (i.e., where the product could be accessed), removal options, replacement options (i.e., how often will the implant be replaced), visibility (once the implant has been inserted), and pain. Each attribute had three options for the participant to choose from (Fig. 2). Each participant received three pizza pieces of each colour coded implant attribute, totalling 24 pizza pieces. The participants could select one option per attribute and construct a round pizza with only eight slices, representing their ideal MPT implant. The participant’s preferences were captured. This was followed by a discussion, following the discussion guide, about participants’ choices and probing to gain a better understanding of the participant’s MPT implant biomedical characteristic preferences.

### *MPT robot*^1^

**Fig. 3:**
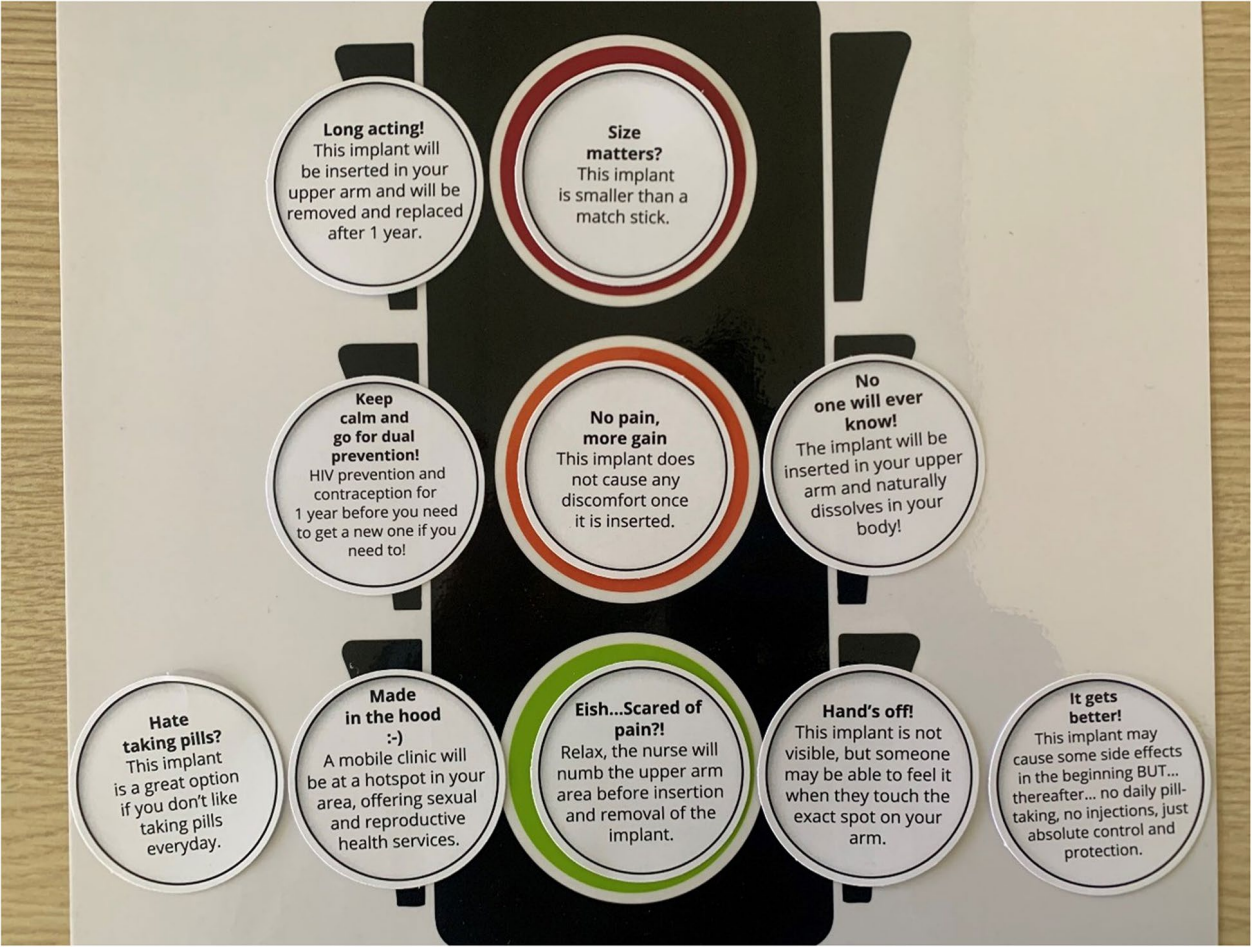
Example of a completed MPT robot activity

 FSWs, due to the nature of their work, had limited time for participation in PAR activities, therefore they participated in an alternative, brief activity (MPT robot). This activity sought to provide FSWs the opportunity to rate various MPT implant biomedical characteristics by their degrees of preference. Participants were given ten tokens and a laminated robot (traffic light) poster. Each token described characteristics of the MPT implant such as functionality, efficacy, side effects, pain associated with insertion or removal, discreetness, and convenience. FSWs were asked to place a token on a robot colour, indicating their degree of preference (See Fig. 3): tokens on the green robot light reflected the participants most desirable MPT characteristics and would promote usage. The orange robot light represented uncertainty about that characteristics and suggested that this characteristic would limit implant usage. Tokens on the red robot light were the most unfavourable MPT characteristics and would prevent usage. A discussion following the discussion guide, then ensued about their choices and views, probing to gain a better understanding of the preferences of each participant when it comes to an MPT implant.

### Data management

The two or three workshop guides from each workshop were transcribed by each observer and consolidated into one workshop guide by the Researcher (PM) or Associate Researcher (NM), totalling 14 workshop guides (six for AGYW, six for women > 24 years and two for FSWs). The 14 workshop guides where then consolidated into one guide per population group (AGYW, women > 24 years and FSW) with data from all three districts. Tally scores from the PAR activities were captured into Microsoft Excel using the completed ballot papers and pictures as the data sources.

### Data analysis

Quantitative descriptive analysis was conducted to determine the frequency and ranking for each demand creation message, channel, and each product and service provision attribute, by population group of interest. A chi-square test was used to assess MPT implant characteristics associated with age. These quantitative results were presented descriptively using distribution tables disaggregated by population group (AGYW, women > 24 years and FSWs). Workshop guides were imported and coded in NVivo V12. One analysis workshop was held where two team members (PM and NM) discussed the emerging data, coding and themes. The first round of codes were created through open coding of one workshop guide during the workshop. The remaining two workshop guides were divided among and coded by PM and NM. Based on the codebook, these qualitative data were then analysed using deductive and thematic analysis to describe and explore the preferences for the different demand creation messages and channels, and preferences for the different MPT implant and service provision attributes.

## Results

There were 373 participants recruited from the fixed clinics and outreach community mobile sites, of these, 253 (67.8%) attended the PAR workshops. Forty seven PAR workshop participants were recruited through snowballing, and three had missing data for place of recruitment. Overall, a total of 303 females participated in the PAR workshops: 104 AGYW, 157 women > 24 years, and 42 FSWs. Overall, 14 PAR workshops were conducted: six with AGYW, six with women > 24 years and two with FSWs. There were 21 participants in each FSW workshop and due to time constraints, only one activity was completed per workshop. One group participated in ‘MPT messaging elections’ and the other completed the ‘MPT robot’.

### MPT messaging elections

Due to time constraints in some workshops, 240 out of 303 workshop participants completed this activity, and only 236 completed the activity as instructed. The data presented below is from the 236 participants: 42.6% were AGYW, 48.5% were women > 24 years and 8.9% were FSW. Overall, the top three messages were, *“I want to be safe, not sorry. I have the power to protect myself!”* (20.6%, number of votes = 146); “*Nothing can stand in the way of me living my best life! I am protected! #MPTlove”* (16.8%, number of votes = 119) and “*Sebenzisa iMPT [Use MPT] to handle two birds with one stone”* (Table 1). Participants said that these messages appealed to them because they were encouraging and empowering. There were minimal differences between AGYW, women > 24 years, and FSWs in the top three messages selected.

**Table 1:** Number of votes per demand creation message, as voted by the study participants^a^

Messages	Frequency (%, number of votes)			
	Total votes (100%, 708)	AGYW (42.8%, 303)	Women > 24 years (48.3%, 342)	FSW (8.9%, 63)
1. I want to be safe, not sorry. I have the power to protect myself!	20.6 (146)	20.4 (62)	20.8 (71)	20.6 (13)
2. Nothing can stand in the way of living my best life! I am protected! #MPTlove.	16.8 (119)	17.2 (52)	16.7 (57)	15.9 (10)
3. Sebenzisa* iMPT [Use MPT] to handle two birds with one stone	15.8 (112)	17.2 (52)	14.0 (48)	19.0 (12)
4. With MPTs …I have choices! I choose what works best for me.	13.7 (97)	14.5 (44)	13.2 (45)	12.7 (8)
5. O ska tseya di chance, bopha nge MPT uzivikele*[Don’t take chances, use MPT to protect yourself against HIV and Pregnancy] against HIV & pregnancy.	13.0 (92)	11.2 (34)	14.0 (48)	15.9 (10)
6. I have no fear when there are so many options to choose from!	8.1 (57)	7.9 (24)	7.9 (27)	9.5 (6)
7. Don’t tatazel, zibambe nge [don’t be afraid, use] MPT!*	6.4 (45)	6.6 (20)	7.0 (24)	1.6 (1)
8. How I choose to prevent HIV and pregnancy, is my business - If I don’t want you to know, you won’t know.	5.6 (40)	5.0 (15)	6.4 (22)	4.8 (3)
**Total votes**	100 (708)^a^	100 (303)	100 (342)	100 (63)

^a^The total number of votes were 3*236 or 708

### Considerations for messaging

#### Positive messaging and language

During the discussion, participants said they preferred messages that were empowering, motivational and encouraged them to be confident in themselves and their ability to make good choices regarding their health and their bodies. They liked that such messages shift their focus from other people and what they will think. Rather, they encouraged them to take control and responsibility of their own health and protect oneself from HIV and unplanned pregnancy. Participants also liked messages that spoke to practising safer sex. Some felt that these would attract young people and encourage them to take the correct measures to prevent HIV and unplanned pregnancy. Participants also highlighted the importance of using colloquial language, including terms in a local language or dialect when developing messaging for specific population groups. There were Zulu and Sesotho^2^ terms used in the messaging examples during the activities and participants who did not know these languages struggled to understand these messages. Therefore, the languages used should be tailored to a specific region. This will ensure that these messages are understood by community members and potential end users who may not understand English or other official languages.

### MPT demand creation channels and tactics

Of the 303 workshop participants, 240 participated in this activity due to time limitations during some workshops and only 235 completed the activity as instructed. The data presented below is from the 235 participants: 42.6% were AGYW, 48.5% were women > 24 years and 8.9% were FSW. There were minimal differences between AGYW, women > 24 years, and FSWs in the top three demand creation channels and tactics selected. The top three demand creation channels and tactics were local community radio station and newspapers(18.4%, number of votes = 130); social media and the internet (18.0%, number of votes = 127) and “information in hospitals and clinics” (16.5%, number of votes = 116) (Table 2).

**Table 2:** Number of votes per demand creation channel and tactic, as voted by the study participants^b^

Demand creation channels/tactics	Frequency (%, number of votes)			
	Total votes (100%,705)	AGYW (42.6%, 300)	Women > 24 years (48.5%, 342)	FSW (8.9%, 63)
1. Everyone should learn about MPTs through the local community radio station and newspapers	18.4 (130)	18.0 (54)	19.0 (65)	17.5 (11)
2. We should use social media and the internet to make MPTs famous!!!	18.0 (127)	19.0 (57)	17.3 (59)	17.5 (11)
3. Information about MPTs should be in hospitals and clinics.	16.5 (116)	15.4 (46)	17.6 (60)	15.9 (10)
4. Posters should be in public areas such as on street light poles, notice boards, shopping centres that share information about MPTs.	12.2 (86)	13.0 (39)	10.5 (36)	17.5 (11)
5. Conversations with community stakeholders such as NGOs, CBOs, schools, TVETs are a great way to introduce MPTs to the community.	11.3 (80)	11.0 (33)	12.6 (43)	6.3 (4)
6. “Health services campaigns” days can encourage the uptake of MPTs when they are available in the community.	10.8 (76)	10.3 (31)	10.8 (37)	12.7 (8)
7. Introducing MPTs through a guardian, mother, daughter, and grandmother dialogue will facilitate a better understanding of MPTs and improve community buy-in.	9.5 (67)	9.3 (28)	9.9 (34)	7.9 (5)
8. MPTs should be introduced in hang-out places e.g. Shisanyama ^a^and netball court	3.3 (23)	4.0 (12)	2.3 (8)	4.7 (3)
**Total votes**	100 (705)^b^	100 (300)	100 (342)	100 (63)

^a^Shisanyama is a place where people can buy meat and have a barbeque, buy alcohol and socialize

^b^The total number of votes were 3*235 or 705

### Considerations for demand creation channels and tactics

#### Accessibility

Participants preferred that information on MPT implants be shared through channels that were easily accessible to potential end-users and their communities at large, including the local community radio station and newspapers, social media, and hospitals and clinics. Participants noted that these channels would be an effective way to reach and educate parents on the MPT implants, possibly motivating parents to support their children’s PrEP journey and potentially help end the stigma around HIV prevention products. These channels are already being used by many to catch up on current issues, and educate themselves on a variety of topics.

Social media was said to be the best channel for reaching young people, while also noting that limited internet data and lack of smartphones may be a barrier to access, hence it would be important to use different channels/tactics to cater for different populations. Facebook, Twitter, Instagram, TikTok, and YouTube were the social media platforms that participants said were best suited for sharing information on the MPT implants. The idea of event days associated with health campaigns appeared suitable for AGYW and FSWs.

#### Fostering conversation on MPT implants

The guardian, mother, daughter and grandmother dialogue was the least preferred demand creation tactic. Many stated they would feel uncomfortable with their mothers or grandmothers present, specifically in relation to asking SRH and HIV prevention related questions. However, a few participants believed that once elders in the community learn about MPT implants from the aforementioned channels/tactics (the local community radio station and newspapers, hospitals and clinics, and social media) they will share this information with other elders including parents of young people. This may lead to a widespread knowledge and understanding of MPTs among elders and foster healthy conversations between young people and their parents on the prevention of HIV and unplanned pregnancy.

#### Enabling environment

Some participants said that they would not go and seek information on MPT implants at hospitals and clinics largely due to healthcare providers’ attitudes, which may at times be judgmental and unkind. Those participants suggested that in this case, information on the implants should be distributed by other, relatable young people at clinics and hospitals. It was also suggested that clinics should work in collaboration with schools to distribute SRH, MPT, and PrEP information at school. Many participants said that shisanyamas were not suitable environments for sharing information on the MPT implants because many patrons are usually there to drink alcohol, relax, or just enjoy themselves and therefore might be less interested in SRH matters. To mitigate this, they suggested hosting events and inviting a famous musician or social media influencer who can then tell patrons about the MPT implants.

#### Suggestions for channels/tactics

Participants also suggested channels and tactics that they thought would better reach women and potentially create demand for the MPT implants. Some participants were open to attending and suggested hosting aerobics and colour run^3^ events where information can be shared with attendees. KCD participants suggested door-to-door campaigns where communities can be educated about MPT implants. Having a television drama with a story line focused on raising awareness around MPT implants was also suggested.

### Build your own MPT pizza

Two hundred and sixteen women completed this activity (AGYW = 103 and women > 24 years = 113). Table 3 shows the participant results.

**Table 3:** Female preferred MPT attributes, *n* = 216

MPT product attribute	Attribute Message	Total overall *N* = 216 (100%)		AGYW *n* = 103 (48%)	Females > 24 years *n* = 113 (52%)	Chi2 *p*-value
		Number of votes	%			
**Body placement**	This MPT implant will be inserted in your upper arm and will naturally dissolve in your body! There will be no need for removal.	80	37.4%	43.7%	31.3%	0.111
	This MPT will be a re-fillable implant that is inserted in your upper arm and will be refilled after one year. It is only inserted once and not removed unless you want it out.	55	25.7%	20.4%	30.4%	
	This MPT implant will be inserted in your upper arm and will be replaced after 1 year.	80	37.4%	35.9%	38.4%	
**Prevention characteristics**	This MPT implant will prevent HIV, pregnancy or STIs for 6 months.	22	10.3%	7.8%	12.5%	< 0.001
	This MPT implant will prevent HIV, pregnancy or STIs for 12 months.	107	50.0%	55.3%	44.6%	
	This MPT implant will prevent HIV, pregnancy or STIs for 24 months.	85	39.7%	36.9%	42.0%	
**Side effects**	This MPT implant may cause very mild side effects for the time you are using it.	20	9.3%	8.7%	9.8%	0.922
	This MPT implant may cause side effects such as nausea, weight gain, irregular menstruation but only while your body gets used to it.	124	57.9%	59.2%	56.3%	
	Every medicine has side effects, you can handle it because the prevention is fantastic.	70	32.7%	32.0%	33.0%	
**Service delivery point**	This MPT implant insertion will be done at a local clinic by a healthcare professional who will require you to visit the clinic for check-ups throughout the year.	135	61.1%	71.8%	54.5%	0.037
	This MPT implant insertion will be done at a mobile clinic in your community by a healthcare professional who will require you to visit the mobile clinic for check-ups.	47	21.3%	16.5%	26.8%	
	This implant will be done in a gazebo at a community hotspot by a healthcare professional and does not require and follow-up visits.	39	17.6%	14.6%	21.4%	
**Removal options**	Should you be unhappy with the MPT implant, a health care professional can remove it any time.	80	37.4%	39.8%	34.8%	0.001
	Once the implant is inserted, you won’t be able to remove it because it dissolves.	65	30.4%	37.9%	23.2%	
	Should you be unhappy with the MPT implant, a health care professional can remove during the first 2 months of use.	68	31.8%	18.4%	43.8%	
**Replacement options**	This MPT implant will prevent STIs AND pregnancy and will need to be replaced every year for continued prevention.	66	30.8%	36.9%	25.0%	0.106
	This MPT implant will only prevent HIV and will need to be re-placed every year for continued prevention.	36	16.8%	16.5%	17.0%	
	This MPT implant will prevent HIV AND pregnancy and will need to be replaced every year for continued prevention.	114	53.3%	45.6%	59.8%	
**Visibility**	This MPT implant will only be felt by yourself or others when the area is pressed.	40	18.7%	22.3%	15.2%	0.180
	This MPT implant will be inserted in your upper arm and will not be visible on the outside so nobody will know it is there.	134	62.6%	62.1%	62.5%	
	This MPT implant cannot be seen but could be felt by yourself or others.	43	20.1%	15.5%	24.1%	
**Pain**	A nurse will numb the area before insertion and removal of the MPT implant, so you will not feel it go in or out, however you may experience some mild discomfort or bruising afterwards.	102	47.7%	49.5%	45.5%	0.409
	There will be slight pain during insertion but no further discomfort after the MPT implant is inserted.	91	42.5%	37.9%	46.4%	
	The pain associated with insertion is similar to that of donating blood with some light bruising.	23	10.7%	12.6%	8.9%	

Each of the MPT product attributes will be described in turn.

#### Body placement

37% (*n* = 80) of participants liked the idea of an MPT implant dissolving (biodegradable) in the body and another 37% liked the idea of it needing to be replaced after one year, rather than the refillable MPT option. Those that liked the biodegradable implant said that it was convenient, with no need to revisit the clinic for removal and also no pain associated with removal. Having the option for implant removal was important: many preferred the non-biodegradable implant as it can be removed at any time, especially if they decided they might want to fall pregnant or react negatively to the implant.

#### Prevention characteristics (duration)

Half of the participants (*n* = 107) preferred an MPT that lasted for one year, with 40% (*n* = 85) preferring an MPT that lasted for two years. Long-term protection was perceived as convenient because they would have fewer clinic visits: this fitted their busy lifestyles. Many felt that two years was too long a duration to commit to. There was a statistically significant relationship between the female age group and prevention characteristics (*p* < 0.001).

#### Side effects

Over half (58%, *n* = 124) accepted that there would be side effects but only while the body gets used to the new medication. Participants were not concerned about experiencing side effects, with many acknowledging that all medication has side effects that usually resolve with time. Many expressed that HIV and pregnancy prevention was more important than side effects. They would like information about the type of side effects and how to manage them.

#### Service delivery point

In terms of service delivery, almost two-thirds (61%, *n* = 135) indicated that they preferred MPT insertion to be done at a local clinic by a healthcare professional, rather than at a mobile clinic (21%) or a gazebo in the community (18%). They liked that the local clinic was in a fixed place so they can access it as needed. Some said that the mobile van was not reliable because it does not always have a consistent schedule, while others preferred it because of its perceived convenience. The mobile van was also reported to have no queues and friendlier nurses than the local clinic. Gazebos were said to be lacking privacy and therefore not suitable for MPT insertion or removal. There was a statistically significant relationship between the female age group and preference for service delivery points (*p* = 0.037).

#### Removal and replacement options

Many participants (37%, *n* = 80) preferred to have the option to have the implant removed at any time, in case of adverse reactions or if they wanted to conceive. 30% (*n* = 65) liked the idea that once inserted, the implant will not be removed because it will dissolve, stating that it would be more convenient to use because they would not have to attend a clinic visit for removal. Lastly, 32% (*n* = 68) preferred that if they were unhappy with the implant, a healthcare professional would be able to remove it in the first two months. There was a statistically significant relationship between female age group and MPT removal options (*p* = 0.001).

### Visibility and Pain

Almost two thirds (63%, *n* = 134) liked that the MPT implant would be discreet and not be visible on the outside of their bodies. Participants expressed that their prevention choices should be private, and they should be able to choose to whom they disclose. Disclosing MPT use varied: most participants said that they would not feel comfortable telling their parents, as they are strict and don’t know they are sexually active; some said they wouldn’t tell their partners that the MPT implant also prevents pregnancy as their partners wouldn’t be happy. Disclosing to friends was also not widely accepted due to a lack of trust. However, some participants noted the positives of disclosure to their parents, partners, and friends, supporting them on their MPT journey and reminding them to attend the clinic. Almost half (*n* = 102) of the participants preferred a nurse to numb the area before insertion because they were afraid of pain.

#### MPT robot

Twenty one FSW participants completed this activity (Table 4). Overall, of the 10 tokens that were rated, the four with the highest green rating were *“Hate taking pills? This implant is a great option if you don’t like taking pills every day”* (85.0%, *n* = 17); *“Made in the hood! A mobile clinic will be at a hotspot in your area, offering sexual and reproductive health services”* (80.0%, *n* = 16); *“It gets better! This implant may cause some side effects in the beginning BUT…thereafter…no daily pill-taking, no injections, just absolute control, and protection”* (65.0%, *n* = 13), and *“Keep calm and go for dual prevention! HIV prevention and contraception for 1 year before you need to get a new one if you need to!”* (60.0%, *n* = 12).

**Table 4:**
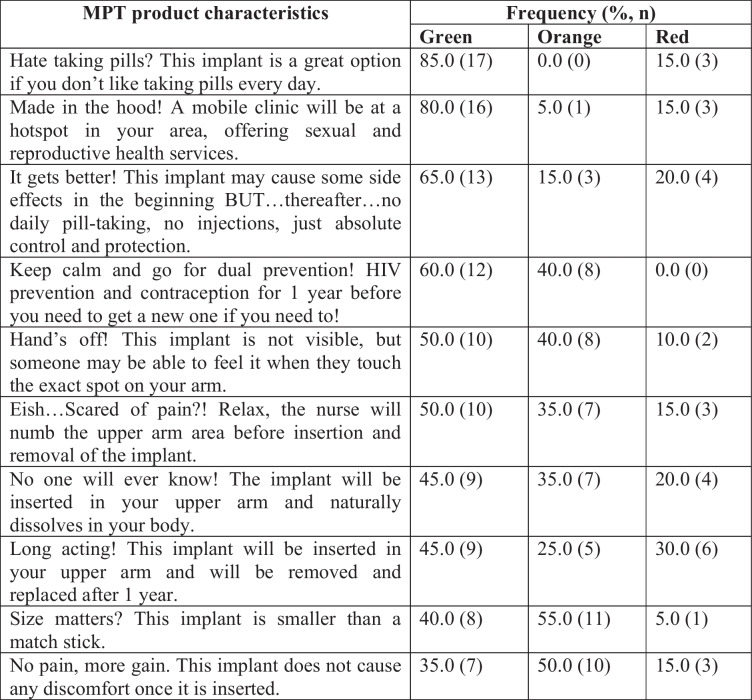
FSW MPT product attribute rating, *n* = 21

### Product characteristics

#### Long-term protection and dual prevention

Most participants did not like taking pills. Due to sex work, they tended to travel a lot, thus sometimes forgetting to take their oral PrEP with them. Therefore, the ability of the MPT implants to provide long-term protection, convenience and discreetness were appealing to them and thus making the implant better suited for their lifestyle. Participants were also excited about the prospect of potentially having an implant that would prevent both HIV and pregnancy, and thus addressing their reproductive health needs with one method.

#### Side-effects

Most participants (65%) said that they would be able to handle the side effects of the MPT implants because all medications have side effects. What mattered the most was not having to worry about remembering to take pills or attending regular clinic visits.

#### Implant removal

Less than half of the participants said they liked that the implant would not require removal. Like the broader group of women, over half of the participants stated that they were concerned about the impact of the implant dissolving in their body, while others were concerned about the potential effects of the implant on their fertility, and not being able to remove the implant if they decide to conceive.

#### Visibility

Participants said that they would not mind if someone touched them and felt the implant. If asked, they would feel comfortable enough to disclose that they are using an MPT implant because a lot of people know of the pregnancy prevention implant so it wouldn’t be something new to a vast majority of people.

#### Pain of insertion and removal

Overall, most participants where not concerned about pain associated with implant insertion or removal. Many were comforted by the idea that the implant area would be numbed before insertion. However, a few participants said that they were not familiar with implant insertion, so they did not know what to expect in terms of pain, while others said they feared pain and needles.

#### Implant size

The small size of the MPT implants was also appealing to the participants as this will keep it discreet. However, some participants were sceptical about the true size of the implant.

### Service delivery characteristics

Most FSW participants preferred receiving the MPT implant from the mobile van than the fixed clinic. They said that the services at the mobile clinic are good, time efficient and make them feel comfortable. These participants liked that the mobile clinic notifies them before coming to their area and it brings services to them. The fixed clinic was less preferred because of long queues, the nurses’ negative attitude, stigma, negative comments from other patients and the fear of being seen by relatives.

## Discussion

This manuscript explored preferences for MPT implant product attributes, educational messaging and demand creation channels and tactics that could improve uptake of MPT implants. The data demonstrates that demand creation messages that were motivational, empowering and encouraged body autonomy were preferred. This is supported by previous research among young South African women who preferred empowering, motivating and simple PrEP messaging [29]. Lessons learned from oral PrEP implementation suggest that demand creation for new products should aim to build user empowerment frameworks that speak to their lifestyles, achieved by working with end users to create these messages [30]. Participants noted that using local languages or dialect would be key to messaging, enabling acquisition and use of information, as supported by previous studies [31, 32]. Community radio stations, newspapers, social media, and information at hospitals and clinics were preferred MPT demand creation channels/tactics by participants. A study in Lesotho also found that preferred sources of PrEP information among participants were television, radio and education sessions at the clinic [33]. Similar findings have been documented in Botswana [34]. Radio remains the most popular mass medium across Africa, despite the increase in cell phones, the growth of social media apps, and streaming music services [35]. In South Africa, more people have access to radios than they do televisions [35]. This may explain the study participants strong preference for community radio stations as a potential channel for generating demand, once MPT implants become available. A study that looked at the use of radio as a tool to encourage and promote HIV/AIDS education among university students found that radio messages were successful in positively influencing personal attitudes and behaviours [36]. Earlier studies also found radio to be instrumental in disseminating health information and building critical knowledge and education [37, 38].

In terms of social media, participants said that Facebook, Instagram, Twitter, TikTok, and YouTube were well suited for sharing MPT information. This is supported by the 30 million active South African social media users > 13 years (2022), all of whom are on Facebook (30 million users), 25.3 million on YouTube, 6.8 million on Instagram, 6.4 million on TikTok and 2.85 million on Twitter [39]. Social media platforms were found to increase PrEP uptake, adherence, and awareness among Black and Latinx women and MSM in America [40]. Evidence of social media interventions as effective platforms in promoting health education in disadvantaged populations has been well documented [41, 42]. As access to cell phones and internet services increases, social media engagement may offer an avenue for more interactive promotion [33]. Common benefits of HIV communication via social media include being able to anonymously engage with content, having access to information and the large geographical reach [43].

Overall there was no resounding preference for a specific demand creation channel and tactic. Therefore, a variety of demand creation channels and tactics should be used to cater for the various needs and lifestyles of the population, to ensure a wide range of people have the potential to hear about new MPT methods. This is supported by previous literature [44]. A tailor-made multi-faceted health campaign is needed to disseminate information about HIV prevention products and encourage the use of these products among those at risk [45]. This will ensure that information on HIV prevention products is easily accessible and raises awareness of the various HIV prevention products available. Continued engagement with the target population when MPT implants become available would be beneficial to ensure they are included in the development of messaging, ensuring it is appealing to the respective groups.

When asked about preferred product attributes, the majority preferred the following: long-term and dual protection, receiving implant services at the local clinic or mobile clinic for the FSWs, minimal side effects, the implant being discreet and private, and minimal insertion/ removal pain due to numbing. Previous studies also found a preference for long-acting products, which provide dual prevention and have minimal side effects [22, 46–48]. Most participants wanted an implant that would prevent both HIV and pregnancy for a long term and the biodegradable nature of the implant was appealing to most. Some who preferred the non-biodegradable implant had concerns about their body’s response to the implant and wanted to have the option to remove the implant anytime. However, previous literature has shown a preference for biodegradable compared to non-biodegradable implants [48, 49]. FSW preferred to receive the implant at a mobile clinic while AGYW and women > 24 years preferred a fixed clinic. This could be attributed to their positive experiences with specialised programs and services [50].

We found varied preferences around MPT implant disclosure. An anticipated benefit of disclosing implant use to parents, friends and partners was support in the form of clinic attendance reminders. Findings from oral PrEP studies have found that the benefits of disclosure after PrEP initiation included social support for PrEP use, adherence reminders, and de-stigmatization of taking PrEP in front of others [51, 52]. Similar findings have been noted in contraception use studies [53, 54]. Community and national sensitization on MPT implants could go some way to limiting the stigma around this prevention method.

The study also found age to be significantly associated with prevention characteristics, service delivery points, and implant removal options, similar to previous research [46]. This reiterates that a one-size fits all approach would not be appropriate to meet the needs of different potential end-user groups. Overall, upholding women’s reproductive health rights includes empowering them to exercise full autonomy over the uptake and discontinuation of their choice of method [55, 56]. This finding highlights the importance of different product formulations for users to choose from based on their lifestyles and preferences [56].

### Strengths and limitations

Our study included perspectives from a diverse group of potential users, including AGYW, women > 24 years, and FSWs. These data were also collected from a variety of geographies, representing different social contexts. The study contributes to formative evidence that may inform MPT implant demand creation however, additional research on community and partner acceptance and suitable service delivery models will need to be conducted closer to product introduction. There was no adjustment for respondent clustering in the statistical analyses which may have biased the results. The demand creation messages and channels/tactics were predetermined by the research team; they were not developed by the participants. Our research explored theoretical product preference: actual preference and potential user views once the implant is developed and available may differ depending on the formulations of the available implant. Future formative studies should undertake studies that seek to understand the demand creation tactics preferred by AGYW (15–17 years) that should be undertaken when new products are developed or under development. The study did not enrol AGYW aged 15 to 17 years therefore their views and needs were not accounted for. Participants were primarily recruited from health facilities, which suggests that they may represent a population that already uses prevention programs and may be different from the overall population. The FSWs were recruited from one site so their views may not be reflective of FSWs from other regions of South Africa.

## Conclusion

Early considerations for women’s product and demand creation preferences are key to product development and implementation strategies that will yield improved uptake of the MPT implant. Educational messaging around the MPT implant should be positive, in local languages, and should convey empowering reasons to try out the MPT implant. This may motivate women to learn more about it and potentially use it. Participants preference for the different demand creation channels varied with a higher preference for community radio stations and newspapers, social media, and hospitals and clinics. As such, a multipronged approach, using many different tactics and channels for demand creation is needed, to reach a wider audience and encourage use, once the MPT implants are developed and are available. This may allow for easier access to information on the MPT implants. Using demand creation channels that allow for engagement with both young and older populations may ensure better reach. Given the appeal of prevention products that provide dual prevention for a long-term with minimal side-effects, MPT implants stand to contribute to the range of HIV and pregnancy prevention options for women.

### Supplementary Information

**Additional file 1.**

## Data Availability

These data that support these study findings are available from the corresponding author on reasonable request.

## Abbreviations

AGYW: Adolescent girls and young women
FSW: Female sex worker
HIV: Human immunodeficiency virus
MPT: Multi-purpose prevention technology
PrEP: Pre-exposure prophylaxis
STI: Sexually transmitted infection
PAR: Participatory action research
KCD: King Cetshwayo District
SRH: Sexual and Reproductive Health

## Footnotes

1 in South Africa, robot refers to traffic lights

2 Zulu and Sesotho are two of the 12 official languages in South Africa.

3 A colour run is a five-kilometre, untimed race. At each kilometre, runners are doused in a different coloured powder. Participants wear white at the starting line and finish the race plastered in colour. Once the race is over, there is a festival with food and music.
