# Supplementary material for: Preferences, educational messaging, and demand creation channels for multipurpose-prevention technologies (MPTs) among women in South Africa
Source: BMC Public Health. 2023 Oct 25;23:2090. doi: 10.1186/s12889-023-16904-0 (PMC10598950; doi:10.1186/s12889-023-16904-0)
Supplement: Supplementary file 1 — Additional file 1. [file 12889_2023_16904_MOESM1_ESM.pdf]

Menu of options for the different pizza slices

This MPT implant will be inserted in your upper arm and will naturally dissolve in your body! There will be no need for removal.

This MPT will be a re-fillable implant that is inserted in your upper arm and will be refilled after one year. It is only inserted once and not removed unless you want it out.

This MPT implant will be inserted in your upper arm and will be replaced after 1 year.

This MPT implant will prevent HIV, pregnancy or STIs for 6 months.

This MPT implant will prevent HIV, pregnancy or STIs for 12 months.

This MPT implant will prevent HIV, pregnancy or STIs for 24 months.

This MPT implant may cause very mild side effects for the time you are using it.

This MPT implant may cause side effects such as nausea, weight gain, irregular menstruation but only while your body gets used to it.

Every medicine has side effects, you can handle it because the prevention is fantastic.

This MPT implant insertion will be done at a local clinic by a healthcare professional who will require you to visit the clinic for check-ups throughout the year.

This MPT implant insertion will be done at a mobile clinic in your community by a healthcare professional who will require you to visit the mobile clinic for check-ups.

This implant will be done in a gazebo at a community hotspot by a healthcare professional and does not require and follow-up visits.

Should you be unhappy with the MPT implant, a health care professional can remove it any time.

Once the implant is inserted, you won't be able to remove it because it dissolves.

Should you be unhappy with the MPT implant, a health care professional can remove during the first 2 months of use.

This MPT implant will prevent STIs AND pregnancy and will need to be replaced every year for continued prevention.

This MPT implant will only prevent HIV and will need to be re-placed every year for continued prevention.

This MPT implant will prevent HIV AND pregnancy and will need to be replaced every year for continued prevention.
